# Patients’ motivations and interest in research: characteristics of volunteers for patient-led projects on PatientsLikeMe

**DOI:** 10.1186/s40900-016-0047-6

**Published:** 2016-12-09

**Authors:** Meaghan Bradley, Julia Braverman, Magdalena Harrington, Paul Wicks

**Affiliations:** PatientsLikeMe, Inc., 160 Second St., Cambridge, MA 02142 USA

**Keywords:** Qualitative research, Patient centeredness, Patient motivation, PRO development, Patient advocacy, PatientsLikeMe

## Abstract

**Plain English summary:**

PLM is an online platform that provides tools for individuals to track their health and connect with other patients and while PLM has invited patients to participate in various research projects throughout the years, an examination into what motivates patients to *want* to get involved in clinical research has not been done. During our analysis of applications submitted by members of the PLM community, we looked for reasons patients want to participate in research and their overall beliefs about clinical research, in general. In addition, we analyzed obstacles and barriers toward patients’ research participation. We observed the following:

- Patients are typically motivated by their individual needs and are most interested in research specific to their own condition.
- To get the most from patients’ involvement and to enhance patients’ contribution towards research goals, researchers should explain the research goal and requirements of each goal in clear and transparent terms, making it easy for patients to understand, thus avoiding any potential miscommunication.

Future studies are needed to determine the best methods for involving patients in clinical research.

**Background:**

Historically, throughout the clinical and medical research arenas, patients have been perceived as passive “subjects” rather than as individuals who may have thoughts regarding research development, research plans, implementation of research studies, and data analysis. However, it is becoming more clear that patients increasingly want to have a more active role in clinical research studies and in the management of their own medical conditions as evidenced by a “no decision about us without us” stance, meaning patients want to make informed decisions about their health while working alongside their healthcare professionals. The central aim of this research study was to determine patients’ motivations for being involved in research design and understand their perceptions of current research practices.

**Methods:**

Two independent qualitative studies were conducted. In Study 1, we analyzed applications submitted by self-identified patients from within the PatientsLikeMe (PLM) community, for acceptance onto our advisory panel. The advisory panel was tasked with developing a best practice guide for how to involve patients in research. During the qualitative analysis, we identified major reasons for and topics of interest associated with PLM members’ motivation to apply to the advisory panel. In Study 2, we analyzed applications from PLM community members and from patients outside the PLM community for a patient-led patient-reported-outcome (PRO) development project. Similar to Study 1, we identified themes associated with patients’ motivations to participate in developing a new PRO.

**Results:**

PLM members are interested in being involved in medical research for various reasons, including facilitating provider-patient communication, improving comprehension of medical information, understanding their disease, and bringing a more individualized approach to health care in general.

**Conclusion:**

Challenges in the process of appropriate involvement of patients in research are discussed. In both studies, the applicants shared their interests in being involved in research. However, in Study 2, many of the patients shared ideas that were not appropriate for the development of a PRO, which indicated limitations in how the invitation and application explained the project to patients. Future studies should contribute to determining the most appropriate method for involving patients in various settings.

## Background

“E-patients” is a term used by Ferguson to describe patients who are “equipped, enabled, empowered, and engaged in their own health care” [1]. It is becoming more often the case that patients living with long-term medical conditions are harnessing Internet-based technologies, such as social networks, crowdsourcing, and citizen science, to access the scientific literature, offer peer support, conduct their own research studies [2], and even develop their own measures to track their health outcomes [3]. PatientsLikeMe (PLM) is a patient-powered research network comprised of over 2,500 condition-specific communities that allows patients with life-changing medical conditions to find other patients with similar conditions, share information about their outcomes, and actively contribute to research. PLM routinely engages its patient community by offering the opportunity to participate in surveys and then uses the data to learn more about these patients and their various conditions. Additionally, PLM members are encouraged to contribute to ongoing research by sharing data about their treatments, symptoms, and outcomes on their profile.

Clinical medical research depends on the willingness of patients to volunteer their time and energy by partaking in experimental interventions, such as clinical trials. However, the traditional medical research paradigm historically used patients as “subjects” – uncritical “laypeople” who did what they were told, were unqualified to provide higher-level input, such as critical appraisal of research methods, and in the worst cases, were victims of unethical manipulation and the denial of accurate information [4]. Even when researchers mean no harm, they often failed to provide study results to patients and, most definitely, did not view them as a true partner in the research process [5]. Medical research has traditionally relied on observable biomarkers and medical professional-interpreted signs or symptoms to assess treatment effectiveness or disease progression. However, this model of clinical medical research is changing, in part due to the decentralized and less hierarchical structure of the Internet as a means to unite patients with similar conditions and to educate them about their condition. When these two existing models come into conflict, there is a risk patients may not want to become involved in clinical research, with consequences for recruitment, attrition, and protocol violations [5].

The cultures of medicine and research are increasingly recognizing the importance of patient involvement throughout the research process. For example, regulatory bodies, such as the FDA, have identified the importance of patient involvement through their patient-focused drug development program [6]. The Patient Centered Outcomes Research Institute (PCORI) is one of the largest funders of patient-focused research, with a specific focus on funding comparative clinical effectiveness research (CER) [7]. PCORI has taken the lead in ensuring patients are actively involved in research, giving them the opportunity to assume such roles as peer reviewers of grant proposals, stakeholders in setting policy, and advisors to ongoing research, allowing patients to stand as empowered peers next to their scientifically- and medically-qualified colleagues [8, 9]. More recently, the focus of medical research has moved toward patient-reported outcome (PRO) measures, defined by the Food and Drug Administration (FDA) as “any report of the status of a patient’s health condition that comes directly from the patient, without interpretation of the patient’s response by a clinician or anyone else” [8]. Unfortunately, many PRO measures have been developed by researchers on the basis of what *they* felt was important to measure rather than what mattered to patients [9].

Despite enthusiasm for patient-centered healthcare and research, the best methods for involving patients in the research process has not yet been established [10]. Many factors need to be addressed and taken into account to ensure the most effective and informative participation of patients in research, including patients’ lack of familiarity with scientific and medical terminology [11]. The success of patients’ engagement in research highly depends on their interest and emotional involvement in the research question and potential outcomes [10]. Hence, involving patients in the early stages of the research process, including the development of research questions, becomes a crucial component of patient-centered research. Indeed, patient involvement in identifying the topics important for research (topic solicitation) is recognized as an essential first step in CER [12]. This project addresses the need to involve patients in topic solicitation, investigates associated barriers and obstacles, and seeks to determine methods to optimize their involvement.

Recognizing the shift towards increased patient involvement in research, PLM established a program to facilitate a patient-led research project through the Open Research Exchange (ORE). ORE, a software platform developed by and integrated with PLM, is an open platform for developing, validating, and sharing health outcome measures that determine issues that are important to patients [13, 14]. ORE has been used by academic and industry researchers in the development of 14 PROs, including measures of treatment burden [14], medication adherence, and suicidal ideation. Researchers involve patients at multiple times during development of a PRO on the ORE (e.g. concept elicitation and soliciting feedback on both individual items and the overall survey) as demonstrated in the creation of a hypertension management instrument [15].

The main goal of these studies was to understand patient perspective and motivation for participating in the development of research study design and policy and also to gain insight into patient perceptions about and potential barriers to their involvement in clinical research. In Study 1, we solicited applications from patient-members within the PLM community to join an advisory panel, whose goal was to provide insight and inform PLM researchers about the patient experience and the needs of patients. The team focused on developing a “best practice guide” for how to involve patients in research studies. An exploratory analysis of the information contained within the applications from patients wanting to be part of the advisory panel was conducted with the goal of describing the major demographic and clinical characteristics of patients who applied, reveal the reasons patients were interested in participating, and determine other motivations for being involved in research.

The goal of Study 2 was to involve patients in developing PRO measurements “from the ground up” to address the needs of other patients like them. For the purpose of this study, we conducted an exploratory content analysis of these applications to discover the key reasons for the patients’ interest in participating in patient-led PRO measurement development. The ultimate goal of these two studies was to learn about patients’ motivation for participating in the research process and to learn about patient expectations and needs while participating in research studies.

## Methods

### Participants

#### Study 1

Patients from within the PLM community who were likely to want to participate in a research process received an invitation (see Appendix B for invitation language and application) to join the advisory panel, specifically including those members who listed ‘Research’ as an interest on their profile, had completed multiple research surveys, or were known by community moderators through earlier involvement in other research projects. Prior to the creation of the advisory panel, members interested in being involved contributed to blogs, videos, or attended conferences alongside PLM. A private message with a link to the application was sent through the PLM website (see Appendix B for invitation language). Additionally, a post in the general PLM forum on the website directed members to the application. The application had no specific role requirements related to previous research experience and it was made clear to potential applicants that a broad range of applicant experience was desired.

#### Study 2

The participation application was made available to both PLM members and non-PLM patients through a press release along with a link to the application on the ORE site (see Appendix C for invitation language and application). Additionally, a post in the general PatientsLikeMe forum on the main website directed PLM members to the application. The press release and the open-access post on the PLM blog were freely republished by other news outlets informing patients about an opportunity to use “your own and others’ experiences to develop a new health outcome measure that is more meaningful, helpful and relevant.”

### Procedures

#### Study 1

Participants had 14 days to respond to the private invitation to join the advisory panel. Following this period, a shortlist of individuals was selected for a follow-up phone screen through a two-wave selection process. The criteria used to determine which patients would receive a follow-up call included that the patient had 1) demonstrated interest, 2) clear communication skills, and 3) provided a description of their experience. In addition, PLM researchers conducted one final review of applicants to ensure that a variety of primary conditions were represented within the sample.

For the purpose of this study, one researcher (MB) completed the exploratory content analysis of a subsample of applications to reveal the most frequent themes describing the motivation behind patients’ participation in the advisory panel and in research, in general. The entire pool of applications was shuffled randomly; applications from both those who were selected to be on the advisory panel and those who were not selected were analyzed. Then, the researcher selected the first 20 applications from the entire pool to review and code, using an inductive approach, and kept selecting the applications until the saturation of themes regarding motivations was reached, i.e., no new themes emerged. For each application, we revealed the key motivation to participate in research and classified the application according to this main theme.

#### Study 2

Criteria for identifying applicants from within the PLM community and patients from outside the PLM community to conduct a phone screen for were 1) clarity of communication, 2) potential for idea to become a patient reported outcome measure, and 3) whether there were enough members within the PLM community with the medical condition of interest. There were no specific requirements related to previous experience with research, condition, and professional background.

For the purpose of this study, one researcher (MB) completed the exploratory content analysis of all applications for the patient-led project to reveal the most frequent themes describing the motivation of patients to develop a PRO measure.

## Results

### Participants

#### Study 1

In total, 5,522 members received the private message invitation; 4,850 of whom, had indicated ‘Research’ as an interest on their profile and 672, of whom, had taken multiple surveys. PLM members submitted a total of 482 unsolicited applications. As a result of the screening process, 84 PLM members were offered phone screens, of whom, 15 were selected and accepted a position on the panel. The following primary medical conditions were represented among members of the panel: attention deficit disorder (ADD), amyotrophic lateral sclerosis (ALS), bipolar II disorder, epilepsy, Fabry’s disease, fibromyalgia, idiopathic pulmonary fibrosis, major depressive disorder, multiple sclerosis, and Parkinson’s disease. Applicants may have also had additional comorbidities although these were not included as a factor in the analyses. A final panel of 14 members (one patient died between acceptance of the invitation and the first meeting) was developed. Any applicants not chosen for the Team of Advisors were invited to be part of a Research Ambassadors program.

The applicants’ ages ranged from 16 to 84 years (mean 53.05 years [SD = 12.33 years]), which made this applicant pool significantly older *(p* < 0.01) than the general PLM population (mean age 46.7 years ± 12.01 years). Overall, 333 (70%) of the applicants were female (5 applicants did not report their gender), which made the proportion of females applying to be on the panel higher than in the general PLM population (54.7%). No information about ethnicity or education was collected.

Applicants reported 95 primary medical conditions in total. The most highly represented conditions (representing 61% of applicants) were multiple sclerosis (17%) and fibromyalgia (15%). Appendix A: Table 1 presents the most frequent primary medical conditions, which were reflective of the distribution of population sizes on the PLM platform. The length of diagnosis ranged from 0 to 52 years across all conditions, with an average of 11.62 (SD = 11.11) years, and a median of 7 years.

The level of prior experience with research varied but the majority of applicants reported their experience focused on informal personal research they performed to learn more about their condition (Appendix A: Table 2). In the sample, 29% had participated in some type of research study, 16% had participated in a clinical trial, and 5% reported having worked professionally as a researcher at the time of the application or in the past.

#### Study 2

Applications were received from 33 participants including 18 PLM members and 15 individuals outside of PLM. Among PLM members, 8 (26%) were women and mean age of all PLM members was 48 ± 14 years (range 26–87 years). Patients from outside of PLM did not declare their age and gender. No additional demographic information was collected.

In total, 33 applications were reviewed for clarity and specificity of idea along with whether the disease condition was strongly represented within the PLM patient population. Three applicants were selected for phone interviews and were invited to continue with the process; only one applicant accepted the project and began the next steps of developing the patient-reported outcome measure.

### Content analysis

#### Study 1

The exploratory content analysis revealed four themes reflecting key reasons for PLM members’ motivation to participate in the advisory panel and the research process. Many applicants did not clearly explain their reason to participate or for their interest in research and these responses were categorized as ‘Unclassified’. The responses were read until the exhaustion of sources, i.e., when no new knowledge was forthcoming. The saturation point was reached after 60 applications; the researcher read 5 more applications to confirm that no new themes emerged. In total, 65 applications were reviewed.

The first theme that emerged revolved around being an equal partner in doctor-patient communications. One response, in particular, was that *“I appreciate having a doctor who treats me as a partner in this process of learning and treatment [of] my health challenges and who is willing to learn from me as well.”* A majority of the members of the advisory board expressed a desire to be treated as equal partners by doctors and researchers. Patients reported feeling their healthcare providers often neglected their voice. They wanted medical researchers and providers see their situation in its entirety, rather than be focused on the single disease.

The second theme was centered around the patient’s desire to understand the cause(s) and nature of their disease. For example, one response was *“understanding and pinpointing what causes multiple sclerosis and how to prevent people from getting it.”* Although some of the other themes contributed to motivation, the most explicitly listed reason for motivation was to understand the cause of disease.

A third theme involved influencing the general approach of healthcare, specifically, the patient’s desire for more effective communication about their condition from their healthcare providers. One patient’s response was that *“information provided in a way that I can understand, in my own language and culture, geared towards my level of health literacy would be a good start.”* Many patients focused on ways to change how healthcare providers and researchers interact with patients. Although patients acknowledge they may have the same diagnosis as someone else, they wanted doctors to approach them as individuals, understanding that what might serve as an appropriate treatment for one patient may not work for another.

The fourth theme was centered around participation in decision making. *One patient’s response was “The obvious perspective is that patients should have some voice in decisions regarding what research should be conducted, what the participants in research should be expected to do, how participants in research should be selected, and how results of research should be communicated.”* Patients felt they had an important voice in all aspects of care and research. They voiced the need for active inclusion in order to know where to start in terms of finding information and learning how to be involved in their own health care decisions. Some patients reported they take a strong advocacy role in their condition and want to help others communicate with healthcare providers. Frequencies of each theme as they appeared in the sample of applications are presented in Appendix A: Table 3.

#### Study 2

The exploratory content analysis of the responses in all applications from patients within the PLM community and those outside the PLM community revealed the occurrence of five themes reflecting the key reasons for patients’ interest in participating in a patient-led PRO measure development project. Applications lacking consistent information about the patient’s interest were categorized as “incomplete information.”

The first theme revolved around healthcare planning needs. One applicant’s response, in particular, was that *“The measure would be used in primary care practices in planning for the youth's transition to the adult health care system.”* The majority of applicants suggested ideas for measurements that would be useful for the healthcare planning needs of a specific population. The measure was not intended for the patient to use themselves for ongoing monitoring of symptoms but rather for a healthcare provider to use for management and planning of a patient’s condition.

The second theme involved treatment outcomes. One particular response was *“I want to measure the effectiveness of a mind-body, touch based intervention such as therapeutic massage for chronic pain.”*, while another suggested *“A survey (either questionnaire or by email or phone contact), to determine how UVB-NB [Ultraviolet B-Narrowband] was implemented in the treatment plan for psoriasis.”* Applicants were interested in research directed towards analyzing the effectiveness of an intervention, such as massage or an electro-stimulator device, for treating chronic pain, for example. They also were interested in understanding disease outcomes related to treatments for disease.

The third theme involved issues of symptom assessment. One applicant responded by *“I would like new standards for measuring pain.”* Twelve percent of the applicants proposed ideas for developing new ways to measure symptoms. These applicants described a symptom or set of symptoms of interest and associated them with a condition of interest.

A fourth theme was a desire to understand the cause of disease, as expressed in one applicant’s response:

*“Dietary contributions to kidney stone formation in young adult females (20–35 years).”* Fifteen percent of the proposed ideas were about understanding the cause of a certain disease or new diagnostic measures to better characterize the disease. These ideas ranged from questioning patient history to understanding exposures or identifying biomarkers indicative of disease.

The fifth and final theme was making the patient the center of care. One applicant responded with *“All patients whose interest is participating more with physicians in a meaningful way, getting results more effectively and efficiently, those wanting control over their own data.”* Under this umbrella, applicants focused on concepts that were essential to making patients the center of care, whether it was giving them a tool to communicate with providers or asking them about their satisfaction with care. Frequencies of each theme as they appeared in the sample of applications are presented in Appendix A: Table 4.

## Discussion

This paper describes two independent research projects both of which aimed to determine patients’ motivations for being involved in research design and to understand their perceptions of current research practices. A patient’s desire to be involved in the research process has been shown to increase their involvement in healthcare and research [16]. Patients can be involved in their own healthcare decisions in various ways, including providing input on treatments, symptoms, or other important aspects of their condition to measure. Additionally, patients provide unique insight into their needs that can help drive the focus of research. Some researchers have started to develop training materials for patients and researchers and by sharing these materials with other researchers and receiving feedback from patients, a standardized set of training materials can be created as a best practice when involving patients in research [17]. Previous studies that involved patients into the research process also emphasized the need to enhance communication to secure effective patient participation in the research process [10, 18]. To receive the most valuable impact from patients engaged in research, researchers and clinicians should make an effort to adequately and transparently describe the research goal and the conceptual framework.

In our first study (Study 1), we demonstrated that patients are, indeed, motivated to participate in healthcare and research and for various reasons. Some patients were mostly motivated to figure out the underlying mechanisms of their disease and wanted to engage with researchers on this topic. As previously shown in the literature, this desire for understanding of disease is central for patients [19]. Other patients noted there were opportunities for educating and empowering the patient population to speak up and demand to be included in the decision-making process in healthcare. Many felt they needed to receive more focused and individualized care that acknowledged how they are different from those around them. This reflects the same need for individualized and carefully targeted care previously emphasized in the literature [20].

Furthermore, patients wanted to become more empowered in issues surrounding their own healthcare but emphasized a need to receive support from healthcare professionals in order to be able to contribute effectively. There were some patients who may be able to empower others who do not feel as capable of making their voice heard. Previous literature confirms this sentiment as ‘empowered, engaged e-patients are a growing social movement’ [21].

Our second study (Study 2) was comprised of both PLM members and non-PLM patients with a goal to discover the key reasons for the patients’ interest in participating in patient-led PRO measurement development. Although many of the applicants presented interesting ideas or demonstrated a concept important to research, the majority of these were deemed not viable for developing a PRO measure by a psychometrician (MH). In addition to the applications with incomplete information, some applications recommended an assessment of an intervention or a way to determine biomarkers to reveal the underlying cause of disease, which were outside the scope of this patient-led PRO project; therefore, the patients who suggested them were not invited to proceed further.

The results of Study 2 revealed significant interest in PRO development across a broad set of topics. Although patients frequently had a difficult time stating what they wanted to measure in a way that would be appropriate for a PRO, many of them were capable of articulating their area of interest. Patients demonstrated a desire for individualized and focused measures specific to their disease. Although in the scope of the given research we were unable to elaborate on and further process many applications, the applicant who worked on the patient-led project was able to point out gaps that could help researchers tailor a PRO measure to better fit her condition, as was demonstrated in previous literature [9]. In an earlier study, a patient living with ALS led the development of modifications to a widely-used existing PRO that suffered from a floor effect, meaning many subjects scored at the bottom of the measure [22]. Therefore, the measure was insensitive to change in patients with the most advanced stages of ALS [3]. By collaborating with researchers, this patient was able to identify and validate new PRO items, which are now in use by the wider research community [3, 9]. In a separate example, researchers examining patient involvement in the Outcome Measures in Rheumatology (OMERACT) conferences determined patient involvement in the conferences impacted the creation of outcome measures. During these conferences, patients have presented new domains important to their experience and influenced the development of core outcome sets [23].

Limitations existed in both of the studies. First, we acknowledge that the data analyzed in both studies was not collected to address the specific research questions, i.e., patients’ motivation to take part in research and patients’ perspective of the current research practice. In both studies, we conducted a secondary analysis of data primarily collected for different purposes. The advantage of this analysis is that patients expressed their motivation and perspective spontaneously. On the other hand, the questions posed to the applicants as part of the process pre-determined the permitted range of responses revealed by the content analysis. Therefore, the results of the studies should be interpreted with caution and used to inform future studies on the topic.

Limitations specific to Study 2 include the fact that no further information was captured from individuals outside the PLM community regarding their source of information about the study. Furthermore, demographic information was not captured from applicants. Although this does not affect validity of the results, obtaining this information in the future may enhance our understanding of the patient population interested in research involvement. Separately, another limitation is some patients might view the invitation as being complex and decide against applying thinking the skillset required is higher than their expertise. The researchers developed open-ended questions in order to give patients an opportunity to share their ideas without imposing any specific research topics. However, looking back at the findings, proposing specific topics to patients may be a more accessible and effective way to involve patients. Future programs that would target involving patients in the PRO development process should build upon the experience reported in this study. For example, the invitation should explain more specifically about the requirements for PRO development and avoid the use of scientific or technical language, which may not be clear to applicants. Although no specific skills or training are required from the patients to be involved, patients would benefit from more complete information about the scope of PRO measurements, including what can and cannot be measured using PROs. Ideally, the invitation message should be structured in a way to prevent patients from sending incomplete, irrelevant, or excessive information.

Other limitations are mostly related to the sample selection and data analysis methodology. An important limitation for both studies is representativeness of the patient population. Although the PLM group is large in number, it is overrepresented by specific disease types and conditions (e.g., neurological disease, such as ALS or multiple sclerosis). In addition, members of the PLM community tend to be more active and more interested in research than patients in general. They also tend to be higher education and are more likely to be white and to be females. This implies the results of the study should not be directly and immediately generalized upon the general patient population. Instead, they should be perceived as an illustration of patient-oriented approach that reveals important pros and cons of patient involvement, identifying some challenges that may be encountered and benefits that can be achieved. More studies should be conducted to develop the best practice of engaging patients into the process of health care in general and PRO development in particular. Additionally, the patients who responded to our applications likely represent the most motivated patients. These patients may not be representative of the activation levels of the general patient population. However, there is no reason to assume their disease experience is substantially different from others with their condition. Including the most active patients in research provides a foundation for increasing patient involvement overall. As more patients realize clinicians and researchers are willing to hear their voice, they are more likely to be involved.

From the methodological perspective, the current studies did not use a formal and systematic means of qualitative analysis. In both studies, a single rater was mostly responsible in the coding of responses and consulted with an external rater as necessary. Additionally, only one theme was assigned to each response, which was intended to focus on the key motivations to participate in research since the questions were not specifically designed for this purpose. We recommend future studies that are specifically developed for this purpose use several raters and qualitative software to confirm our findings. Future studies should also evaluate the factors that may enhance patient involvement in research outside of an online forum such as patient advocacy programs and training [24, 25].

## Conclusion

The breadth of information found in qualitative review of the applications was extensive and indicated there should be more efforts to involve patients. As shown through each study, involving patients in research is a promising area and should continue to be evaluated. The themes revealed patients are most interested in areas of research directly applicable to their individual condition and cause of disease. Additionally, patients new to being involved in research need to be properly paired with researchers or patients with previous research experience to receive support. Finally, researchers looking to involve patients should be mindful of language used to limit scientific terminology and encourage patients to share what is most important. Next steps in patient involvement should focus on methods for continuous involvement to limit the burden on both patient and researcher while leading to insights able to be incorporated into research.

## Appendix A

**Table 1:** Most frequent (*N* = 10) primary conditions among advisory panel applicants

Primary condition	Frequency	Percent (%)
Multiple Sclerosis	81	16.80
Fibromyalgia	70	14.52
Parkinson’s Disease	52	10.79
Epilepsy	26	5.39
Rheumatoid Arthritis (RA)	19	3.94
Type 2 Diabetes	17	3.53
Amyotrophic Lateral Sclerosis (ALS)	15	3.11
Major Depressive Disorder	15	3.11

**Table 2:** Frequency of research type in analyzed sample of advisory panel applications

Research Type	Frequency
Informal researcher	48%
Study participant	29%
Clinical trial participant	16%
Professional researcher	5%

**Table 3:** Frequency of themes in the analyzed sample of advisory panel applications

Theme	Frequency (%)
Understanding the causes and nature of the disease	18 (28%)
Influencing the general approach of healthcare	17 (26%)
Participating in decision making (having their voice heard)	12 (18%)
Being an equal partner in doctor-patient communication	8 (12%)
Unclassified	10 (15%)

**Table 4:** Frequency of themes in the analyzed sample of patient-led project applications

Theme	Frequency (%)
Healthcare planning needs	8 (24%)
Treatment outcomes	6 (18%)
Symptom assessment	4 (12%)
Understanding disease	5 (15%)
Patient focused care	2 (6%)
Incomplete information	8 (24%)

## Appendix B

### Advisory panel invitation message

I'm really excited to announce that we’re forming our very first team of patient advisors at PatientsLikeMe. We’re looking for 6–12 people for the first year—and I think you could be one of them!

This year, the team will be helping to make research more patient-centric. Since you’re someone who is interested in research, it’s clear you’re as passionate as we are about bringing the patient voice to research efforts. That’s why I’m extending an invitation to you to apply to be on the 2014 team of advisors.

No formal science training is required to be an advisor — your “expertise” is the experience you already have of being a patient. As an advisor, you will work with other team members on initiatives such as:

- Creating a “guide” that highlights new standards for researchers to better engage with patients like you
- Giving regular feedback to researchers on how their work can be more beneficial to patients
- You can apply to be an advisor here [link to application]. Applications are due by April 15.
- I’m really excited about this new opportunity and the impact it can have. I hope to read your application soon!

### Advisory panel application form

- Have you ever volunteered or worked with health-related organizations, research associations, or advocacy groups? (If yes, please tell us what you did and why it was, or was not, satisfying).
- What has been your experience with research? This includes participation in studies (such as clinical trials), doing research on your own or professionally, or as consumer/reader of research.
- What does patient-centeredness (in healthcare and/or research) mean to you?
- In your opinion, what is the most important research question that needs to be addressed in order to better understand your condition?
- What are the barriers to undertaking this research question? How would you address these barriers?
- Is there anything else you’d like to tell us about yourself?

## Appendix C

### Patient-led PRO development project invitation message

Now YOU can lead the next research project on ORE

Do you have an idea for creating a better way to measure disease? If so, we’d love to work with you!

We’ve just opened up ORE to patient-led research initiatives, so that you can develop, test and validate new measures. It’s a whole new way to think about medicine. That’s because health measures are typically developed by doctors and scientists, people who aren’t necessarily living with the disease. So, their measures don’t always assess health or quality of life in way *that matter most to you – the patients.*

Interested?

We’re supporting just two patient led projects this year, so if you’re interested, apply now! Start by answering five questions about what you want to measure [link to application].

### Patient-led PRO development project application

- **What do you want to measure?** Is it about symptoms, or pain, or satisfaction with treatment, for example? It’s best to narrow down your idea to one short sentence, and to be very specific.
- **Why do you want to develop a PRO measure?** Think about why it’s important to you, how useful it would be, and to whom. Have you talked to other patients about their experience? When you can clearly articulate who the measure would most benefit and why, you’ll be clearer about how to create it.
- **Who do you want to test your measure with?** Adults with a chronic condition or women with MS are two examples of populations you could survey.
- **How will your measure be used?** Who will ultimately use it, when, and how often?
- **Are there any other similar measures?** Take some time to review the scientific literature. That’s part of the instrument development process and helps you learn about the topic, develop your own opinion based on previous research, and build on work that has been already done.
- **What are the development stages for your instrument?** They can include concept elicitation, cognitive interviews, test and re-test phase, and a follow up phase. Depending on your project goals, you may incorporate all or only some of these stages.
